# Co-infection of *Plasmodium falciparum* and *Schistosoma mansoni* is associated with anaemia

**DOI:** 10.1186/s12936-023-04709-w

**Published:** 2023-09-14

**Authors:** Sylvester Donne Dassah, Kingsley Enock Nyaah, Dodzi Kwaku Jnr Senoo, Juventus B. Ziem, Yaw Aniweh, Lucas Amenga-Etego, Gordon A. Awandare, James Abugri

**Affiliations:** 1 Department of Biochemistry and Forensic Sciences, School of Chemical and Biochemical Sciences, C. K. Tedam University for Technology and Applied Sciences, Navrongo, Ghana; 2 Navrongo Health Research Centre, Navrongo, Ghana; 3 School of Medicine, C. K. Tedam University for Technology and Applied Sciences, Navrongo, Ghana; 4 West African Centre for Cell Biology of Infectious Pathogens (WACCBIP), Department of Biochemistry, Cell and Molecular Biology, College of Basic and Applied Sciences, University of Ghana, Legon, Ghana

**Keywords:** Malaria, *Plasmodium*, *Schistosoma mansoni*, Intestinal schistosomiasis, Anaemia, Coinfection, School children, Tono irrigation, Ghana

## Abstract

**Background:**

Malaria and schistosomiasis persist as major public health challenge in sub-Saharan Africa. These infections have independently and also in polyparasitic infection been implicated in anaemia and nutritional deficiencies. This study aimed at assessing asymptomatic malaria, intestinal *Schistosoma* infections and the risk of anaemia among school children in the Tono irrigation area in the Kassena Nankana East Municipal (KNEM) in the Upper East Region of Northern Ghana.

**Methods:**

A cross sectional survey of 326 school children was conducted in the KNEM. Kato Katz technique was used to detect *Schistosoma* eggs in stool. Finger-prick capillary blood sample was used for the estimation of haemoglobin (Hb) concentration and blood smear for malaria parasite detection by microscopy.

**Results:**

The average age and Hb concentration were 10.9 years (standard deviation, SD: ± 2.29) and 11.2 g/dl (SD: ± 1.39) respectively with 58.9% (n = 192) being females. The overall prevalence of infection with any of the parasites (single or coinfection) was 49.4% (n = 161, 95% confidence interval, CI [44.0–54.8]). The prevalence of malaria parasite species or *Schistosoma mansoni* was 32.0% (n = 104) and 25.2% (n = 82), respectively with 7.7% (n = 25) coinfection. The prevalence of anaemia in the cohort was 40.5% (95%CI [35.3–45.9]), of which 44.4% harboured at least one of the parasites. The prevalence of anaemia in malaria parasite spp or *S. mansoni* mono-infections was 41.8% and 38.6%, respectively and 64.0% in coinfections. There was no statistically significant difference in the odds of being anaemic in mono-infection with malaria (OR = 1.22, 95% CI 0.71–2.11, p = 0.47) or *S. mansoni* (OR = 1.07, 95% CI 0.58–1.99, p = 0.83) compared to those with no infection. However, the odds of being anaemic and coinfected with malaria parasite species and *S. mansoni* was 3.03 times higher compared to those with no infection (OR = 3.03, 95% CI 1.26–7.28, p = 0.013).

**Conclusion**

The data show a high burden of malaria, *S. mansoni* infection and anaemia among school children in the irrigation communities. The risk of anaemia was exacerbated by coinfections with malaria parasite(s) and *S. mansoni*. Targeted integrated interventions are recommended in this focal area of KNEM.

## Background

Malaria and schistosomiasis both constitute a serious public health challenge and are the topmost parasitic infections causing a significant amount of morbidity and mortality globally [1–4]. In sub–Saharan Africa (SSA), where more than 90% of these illnesses occur, concurrent infections of these parasites are frequently reported [5, 6]. They are especially frequent in rural areas and are intimately linked to poverty [4, 7, 8]. These infections are often common and a significant public health concern in Ghana, particularly among school-age children [9–12]. Malaria, caused primarily by *Plasmodium falciparum*, is prevalent across Ghana at varied levels of endemicity. In the Kassena Nankana East Municipal in the Upper East Region of Northern Ghana, malaria transmission is highly seasonal [13, 14].

In Ghana, *Schistosoma* infections are also common and widespread with an estimated country-wide schistosomiasis prevalence of 23.3% and focal prevalence levels exceeding 50% in some areas [15]. The life cycle of *Schistosoma* parasites revolves around freshwater snails as an intermediate host, which releases the infective form (i.e., cercariae) into fresh water, leading to human infection through contact with the contaminated water. *Schistosoma* infection in most parts of SSA and in Ghana is either intestinal, caused by *Schistosoma mansoni* or urogenital caused by *Schistosoma haematobium* [4, 15]. In the Tono irrigation area in the Kassena Nankana District, *S. mansoni* and *S. haematobium* have been previously reported [16]. The prevalence of *S. mansoni* was found to be 54.2% in the south-western corridor of the district where irrigation canals with intermittent reservoirs supply water from the Tono dam to irrigate farmlands downstream in communities close to the irrigation scheme, with childhood activities making it easy for contact with snail infested stagnant waters and increasing the risk of infections.

Malaria and schistosomiasis collectively and independently have been implicated in nutritional stress among the people they infect [17–21]. Such concurrence of parasitic infections often results in anaemia with serious consequences. Anaemia, a red blood cell disorder characterized by lower-than-normal red blood cell count and insufficient haemoglobin levels, affects approximately 23% of the population worldwide [22]. In SSA, nearly 60% of all children are reported to be anaemic ranging from 23.7% in Rwanda to 87.9% in Burkina Faso [23]. Anaemia alone has been associated with increased mortality in young children [23, 24], with lower cognitive performance [25, 26], and in severe cases, lower aerobic exercise capacity and heart failure [27, 28]. Anaemia and malnutrition increase the risk and severity of infections among the affected individuals and hence, are major causes of death especially among children and pregnant women [21, 23]. The synergistic occurrence of parasitic infections, anaemia and malnutrition exert a negative effect on growth and development of the affected persons [21], 29].

Malaria leads to lower concentration of Hb levels and anaemia through mechanisms such as the destruction of infected red blood cells (RBCs), reduction in non-infected RBCs life span and suppression of bone marrow resulting in decreased erythropoiesis [30–32]. Schistosomes infections has been thought to induce anaemia through extra-corporeal loss resulting in iron deficiency, sequestration in spleen, autoimmune haemolysis and inflammatory mechanisms [33].

To mitigate the effect of these parasitic infections, in Ghana, malaria control programmes are implemented including the use of insecticide-treated bed nets (ITNs), indoor residual spraying (IRS), and a test and treat policy for febrile illnesses with artemisinin-based combination therapy [34, 35]. Mass drug administration (MDA) with praziquantel (PZQ) for *Schistosoma* infection control has been implemented since 2008 mainly among school-age children (SAC) with water, sanitation and hygiene (WASH) campaigns to improve hygiene practices [36]. Despite these interventions, these parasites persist and are endemic in Ghana. School children resident along the Tono irrigation scheme are particularly at high risk as the irrigation canals, reservoirs and stagnant waters in paddy rice fields serve as breeding site for malaria vector mosquitoes and freshwater snails that harbours and transmit intermediate forms of intestinal schistosomiasis [16]. This study was conducted to estimate the current prevalence of malaria, intestinal schistosomiasis, anaemia and the effect of malaria and intestinal schistosomiasis on anaemia in SAC in the Tono irrigation area, Navrongo, in the Upper East Region of Ghana to guide control interventions.

## Methods

### Ethical considerations

Ethical approval for the use of human participants in this study was obtained from the Institutional Review Board of the Navrongo Health Research Centre. Written informed consent was obtained from parents' or guardians of student participants attending the selected schools. Additionally, for children who were ≥ 10 years, an additional assent was obtained. Permission was obtained from the District Education Office and from the Headteachers of the selected schools. Children who tested positive for *S. mansoni* infection, and had low haemoglobin were referred to the C. K. Tedam University Hospital, Navrongo for further evaluation and management. According to the current Ghana Health Service guidelines, asymptomatic infections are not eligible for treatment. However, children who tested positive for malaria were advised to visit the nearest Health Centre for further evaluation and treatment as per Ghana Health Service protocols if they go on to develop fever.

### Study design and area

**Fig. 1:**
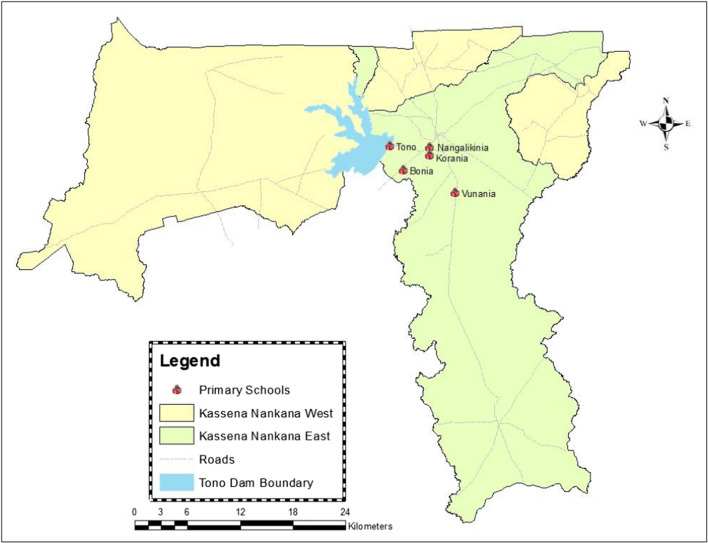
A map showing Kassena-Nankana East and West Districts, the Tono irrigation dam and the location of the schools where the children were recruited

Between September and October 2022, a cross sectional survey was conducted in schools located in five communities (Tono, Nangalikinia, Bonia, Korania and Gaani) in the Kassena Nankana East Municipal (KNEM) located either close to the Tono dam or irrigated lands (Fig. 1). School-age-children (SAC) surveyed from 27th September to 20th October 2022 to determine the association between intestinal schistosomiasis, malaria parasitaemia carriage and anaemia. The KNEM is predominantly a farming community in the Upper East Region of Northern Ghana with an approximate population of 134,000 inhabitants. The main occupations of the inhabitants are subsistence farming and rearing of ruminants. The average annual rainfall in the municipality is about 850 mm and occurs almost entirely between the months of June and October [37]. There is one major dam, the Tono irrigation dam (Fig. 1), and several smaller dams and dug-outs dotted around the study area, which provide water for vegetable cultivation and for animals during the long dry season. These dams and stagnant water from the irrigation canals and reservoirs serve as breeding sites for mosquitoes and freshwater snails that harbour and transmit malaria and intestinal schistosomiasis, respectively.

### Recruitment of study participants

**Fig. 2:**
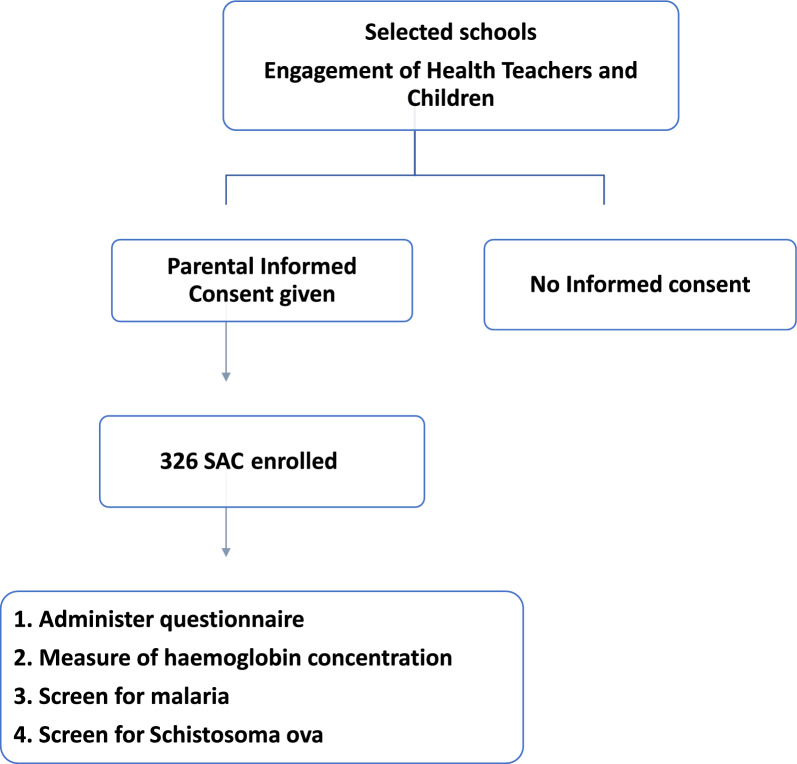
Study flowchart of recruitment procedure and enrolment criteria. *SAC* school-age children

Participants were recruited from all five primary schools within the KNEM located in communities along the Tono irrigation scheme. Each time a school was visited, the study protocol and rationale were explained and only children resident in the area for at least the past six months were eligible for recruitment. Informed consent was obtained from the parent/guardian and prior to sample collection and recruitment. Participants’ recruitment and enrolment is illustrated by Fig. 2. A structured questionnaire was used to capture demographic information and water, sanitation and hygiene (WASH) activities. Stool sample and capillary blood were obtained from all consenting participants.

### Administration of questionnaire

Demographic information, sanitation and water contact activities (bathing, washing clothes, swimming in streams) were collected using a structured questionnaire. The SAC were also asked if they saw blood in their stool in the past days to few weeks. All completed data forms were manually checked for completeness and inconsistencies. The data was then entered into EpiData version 3.1.

### Determination of haemoglobin concentration and malaria parasitaemia

Haemoglobin (Hb) concentration was determined in the field using a portable Urit-12 haemoglobin meter (URIT Medical Electronic Co., Ltd, China) as per the manufacturer’s instructions. In brief, a drop of capillary blood obtained from a finger prick was collected to fill the microcuvette and inserted into a well-calibrated photometer. The haemoglobin concentration was measured in g/dl and recorded instantly.

In addition, thin and thick blood films were also prepared from the finger prick capillary blood and allowed to air dry. The prepared slides were transported to the C. K. Tedam University Hospital laboratory. The thin film was fixed with methanol for a few seconds. Both blood films were then stained with 10% Giemsa stain for 15 minutes after which they were gently rinsed with buffered tap water for about 10 seconds and allowed to air dry [38]. The stained blood films were examined under light microscope using × 100 high power magnification under oil immersion. On the thick films, the trophozoites and/or gametocytes stages of the malaria parasites were identified and counted against 200 White Blood Cells (WBCs). Parasite speciation was done using the thin films. Negative results were assigned after examining 200 high-power fields of the thick film at × 100 magnification [38].

### Stool analysis for *S. mansoni* ova by the Kato-Katz technique

Stool samples collected were transported to the CKT-UTAS university hospital laboratory, Navrongo, in a pre-chilled cold box with ice packs (2–8 °C) for analysis. From each sample, Kato-Katz thick smear was prepared according to the techniques previously described by Katz et al*.,* 1972 [39]. In brief, a template that holds about 41.7 mg of homogenized stool sample was used to prepare each slide with pre-socked cellophane in malachite green glycerol solution laid over it and pressed to give a uniform paste. The slides were examined to identify *S. mansoni* eggs and other intestinal parasites.

To ensure quality control, 10% of the Kato-Katz thick smear slides and those of malaria thin and thick smears were crossed examined by two independent experienced microscopists from the Navrongo Health Research Centre.

### Data analysis

Data analysis was done using GraphPad Prism (version 9.0.0) and STATA (version 17.0) statistical software. Malaria parasite counts were converted to parasite density/μL of blood assuming 8000 WBCs/μL of blood. Malaria parasite intensity was classified as light infection (1–499 parasites/μl of blood), moderate infection (500–1999 parasites/μl of blood) and heavy infection (≥ 2000 parasites/μl of blood) [20]. To obtain the number of *S. mansoni* eggs per gram (Epg) of stool, the egg counts were multiplied by 24 standard constants. The intensity of *S. mansoni* infection was considered light, moderate, and heavy if the egg load was 1–99 Epg, 100–399 Epg, and ≥ 400 Epg, respectively [40]. For quality control purposes, 10% of the slides were randomly selected and re-examined by a senior microscopist at the Navrongo Health Research Centre. Haemoglobin concentrations were grouped according to the severity of anaemia, as Hb < 7.0 g/dl (severe anaemia), 7.0 to  < 11.0 g/dl (moderate anaemia) and ≥ 11.0 g/dl (normal) [41]. Infection status was grouped into: (i) no infection, (ii) malaria only, (iii) *S. mansoni* only; and (iv) coinfection of malaria parasite species and *S. mansoni*.

The Chi-square test (*χ*^2^) or Fisher Exact test was used to compare proportions of categorical variables such as sociodemographic factors, prevalence of malaria infection, *S. mansoni* infection and anaemia. To allow for estimations of the odds ratio between the infection groups and anaemia, haemoglobin concentrations were categorised into normal, moderate and severe anaemia. To investigate predictors of anaemia, a logistic regression model was fitted. To evaluate the association of SAC’s activities and demographic factors with the risk of *S. mansoni* infections, a separate logistic regression model adjusting for the location of schools was implemented. Estimates were considered statistically significant with an alpha value less than 0.05.

## Results

### Descriptive characteristics of study participants

A total of 326 primary school children were screened in this study for malaria and *S. mansoni* infections and the presence of anaemia. The average age was 10.9 years (standard deviation, SD: ± 2.29; range: 5–17). The basic demographic characteristics of the study participants are presented in Table 1. Out of the total, 58.9% (n = 192) were females and 41.1% (n = 134) were males. The children were grouped into three age categories with more than half of the children (n = 184, 56.4%) within the age group, 9–12 years. There were significant differences between the reported occupation of parents across the location of the schools surveyed (*χ*^2^ test, p < 0.001), with the majority being farmers (n = 226, 73.62%). The source of drinking water for most of the participants was tap or borehole water (n = 316, 97.2%). Open bush defecation was prevalent (n = 296, 91.4%) in the area and similar across the sites.

**Table 1:** Socio-demographic parameters of study participants

Characteristics	Bonia	Gaani	Korania	Nangalikinia	Tono	Total	p-value
	n (%)	n (%)	n (%)	n (%)	n (%)	n (%)	
Gender							
Male	27 (20.15)	22(16.42)	21 (15.67)	32 (23.88)	32 (23.88)	134 (41.10)	0.18
Female	35 (18.23)	47 (24.48)	40 (20.83)	37 (19.27)	33 ()17.19	192 (58.90)	
Total	62 (19.02)	69 (21.17)	61 (18.71)	69 (21.17)	65 (19.94)	326 (100.0)	
Age group (years)							
5–8	17 (30.91)	7 (12.73)	9 (16.36)	9 (16.36)	13 (23.64)	55 (16.87)	0.014
9–12	34 (18.48)	33 (17.93)	34 (18.48)	42 (22.83)	41 (22.28)	184 (56.44)	
13–17	11 (12.64)	29 (33.33)	18 (20.69)	18 (20.69)	11 (12.64)	87 (26.69)	
Guardian/parent occupational status							
Artisan	0 (0.00)	0 (0.00)	4 (36.36)	7 (63.64)	0 (0.00)	11 (3.58)	< 0.001
Civil servant	2 (10.00)	4 (20.00)	8 (40.00)	0 (0.00)	6 (30.00)	20 (6.51)	
Farmer	50 (22.12)	55 (24.34)	35 (15.49)	48 ()21.24	38 (16.81)	226 (73.62)	
Others	9 (18.00)	3 (6.00)	13 (26.00)	12 (24.00)	13 (26.00)	50 (16.29)	
Source of drinking water							
Tap/borehole	60 (18.99)	67 (21.20)	58 (18.35)	69 (21.84)	62 (19.62)	316 (97.23)	0.35
Bottle/sachet	0 (0.00)	0 (0.00)	1 (33.33)	0 (0.00)	2 (66.67)	3 (0.92)	
Dam/stream	1 (33.33)	1 (33.33)	0 (0.00)	0 (0.00)	1 (33.33)	3 (0.92)	
Well	0 (0.00)	1 (33.33)	2 (66.67)	0 (0.00)	0 (0.00)	3 (0.92)	
Type of toilet facility							
Open bush defecation	60 (20.27)	60 (20.27)	55 (18.58)	65 (21.96)	56 (18.92)	296 (91.36)	0.13
Pit latrine	2 (11.11)	7 (38.89)	3 (16.67)	3 (16.67)	3 (16.67)	18 (5.56)	
Water closet	0 (0.00)	1 (10.00)	3 (30.00)	1 (10.00)	5 (50.00)	10 (3.09)	
Visit or use dam/stream							
No	27 (24.11)	16 (14.29)	29 (25.89)	14 (12.50)	26 (23.21)	112 (34.46)	0.001
Yes	35 (16.43)	53 (24.88)	31 (14.55)	55 (25.82)	39 (18.31)	213 (65.54)	
Activities performed in the dam/stream							
Swimming/bathing	28 (23.33)	11 (9.17)	22 (18.33)	29 (24.17)	30 (25.00)	120 (56.87)	< 0.001
Domestic	6 (16.67)	0 (0.00)	7 (19.44)	17 (47.22)	6 (16.67)	36 (17.06)	
Farming	0 (0.00)	43 (78.18)	0 (0.00)	9 (16.36)	3 (5.45)	55 (26.07)	
Knowledge of Bilharzia							
No	60 (22.39)	42 (15.67)	41 (15.30)	64 (23.88)	61 (22.76)	268 (82.21)	< 0.001
Yes	2 (3.45)	27 (46.55)	20 (34.48)	5 (8.62)	4 (6.90)	58 (17.79)	
See blood in stool							
No	46 (20.81)	46 (20.81)	42 (19.01)	42 (19.01)	45 (20.36)	221 (67.79)	0.59
Yes	16 (15.24)	23 (21.90)	19 (18.10)	27 (25.71)	20 (19.05)	105 (32.21)	
See blood in urine							
No	58 (19.66)	55 (18.64)	58 (19.66)	66 (22.37)	58 (19.66)	295 (90.77)	0.017
Yes	4 (13.33)	13 (43.33)	3 (10.00)	3 (10.00)	7 (23.33)	30 (9.23)	

Chi-square test (*χ*^2^) or Fisher Exact test was used to compare the proportions of categorical variables

n, number of participants; %, percentage

Overall, 65.5% (n = 213) of the children in the area had access to the canals or used the dam for activities, such as bathing/swimming (n = 120, 56.9%), domestic purposes (n = 36, 17.1%) and farming (n = 55, 26.1%). Among the participants, 17.8% (n = 58) knew about schistosomiasis and its prevention measures, and 32.2% (n = 105) and 9.2% (n = 30) reported having previously seen blood in their stool or urine respectively from days to few weeks prior to the study.

### Malaria parasite carriage and density

**Fig. 3:**
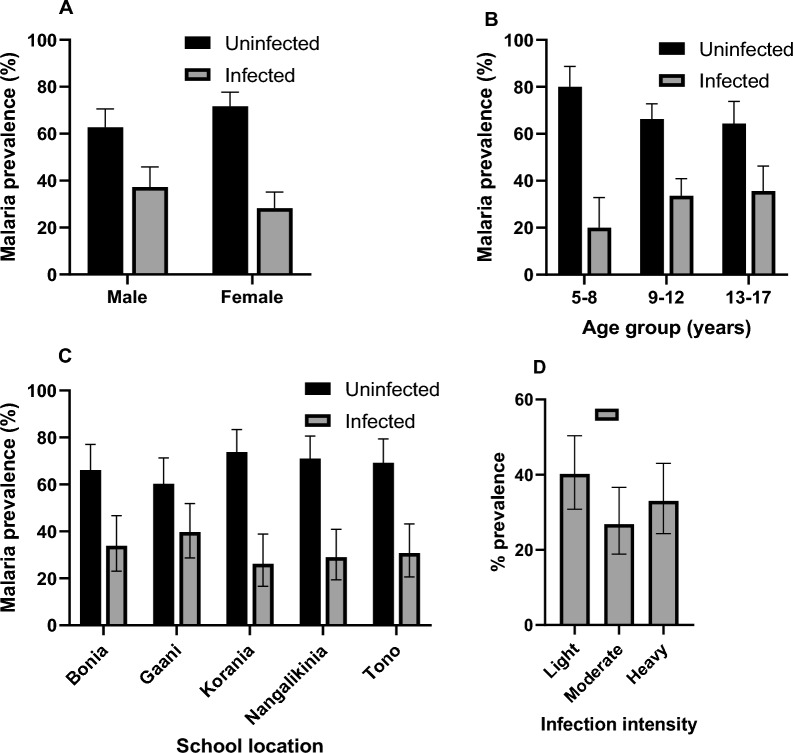
Prevalence of malaria parasite carriage and infection intensity. The prevalence of malaria was stratified by gender (**A**), age groups (**B**), and by communities where schools were located (**C**). The infection intensity was classified as light infection (1–499 parasites/μl of blood), moderate infection (500–1999 parasites/μl of blood) and heavy infection (≥ 2000 parasites/μl of blood) (**D**). The bar chart represents the percentage (%) prevalence with 95% confidence intervals (i.e., error bars). The dark (black) bars are the uninfected group and the light bars represent the infected group

Malaria parasite carriage was 32.0% (95% CI 27.1–37.3) with *Plasmodium falciparum* (98%) being the predominant malaria parasite species detected. In few cases, *Plasmodium malariae* (1%) and mixed infection of *P. falciparum* and *P. malariae* (1%) were also detected. The prevalence of malaria parasite carriage was higher in males, 37.3% compared to females, 28.3% but the difference was not statistically significant (*p* = 0.085) (Fig. 3A). The parasite carriage rate increased from 20.0% among children aged 5–8 years old to 35.6% among those aged 13–17 years old (Fig. 3B) but the difference was not statistically significant. The variation of parasite carriage rate among the schools (26.2% in Korania to 39.7% in Gaani) was not significantly different (Fig. 3C). Parasitaemia was light in 40.2% (39/104), moderate in 26.8% (26/104) and heavy in 33.0% (32/104) of the children (Fig. 3D). More males (42.9%) than their female (21.3%) counterparts tend to harbour heavy parasite load but this was not statistically significant (Table 2). Younger children were observed to significantly (*p* = 0.003) carry heavy (81.8%) infections compared to older children (14.8%).

**Table 2:** Malaria infection intensity by gender and age group

	Light		Moderate		Heavy		p-value
	n	% (95%CI)	n	% (95%CI)	n	% (95%CI)	
Gender							
Male	18	36.7 (23.4–51.7)	10	20.4 (10.2–34.3)	21	42.9 (28.8–57.8)	0.065
Female	21	44.7 (30.2–59.9)	16	34.0 (20.9–49.3)	10	21.3 (10.7–35.7)	
Age group							
5–8 years	1	9.1 (0.2–41.3)	1	9.1 (0.2–41.3)	9	81.8 (48.2–97.7)	0.003
9–12 years	24	41.4 (28.6–55.1)	16	27.6 (16.7–40.9)	18	31.0 (19.5–44.5)	
13–17 years	14	51.9 (31.9–71.3)	9	33.3 (16.5–54.0)	4	14.8 (4.2–33.7)	

Chi-square test (*χ*^2^) was used to compare the proportions of categorical variables

n, number of participants; %, percentage; 95% CI, 95% confidence interval

### *Schistosoma mansoni* prevalence and infection intensity

**Fig. 4:**
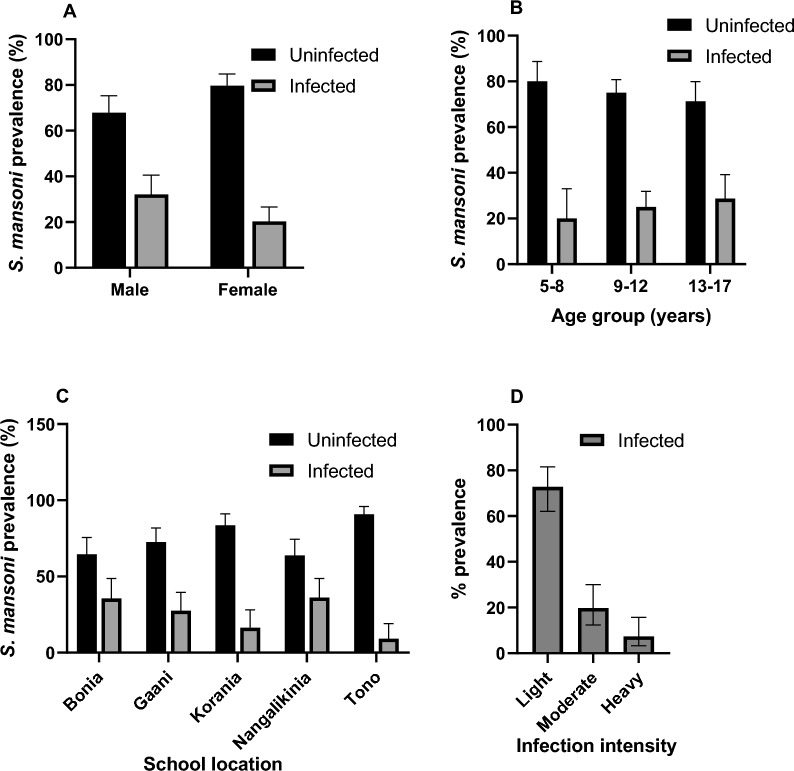
*Schistosoma mansoni* infection prevalence and intensity. The prevalence of *S. mansoni* was stratified by gender (**A**), age groups (**B**), and by communities where schools were located (**C**). The *S. mansoni* infection intensity was classified as light infection (1–99 eggs/gram), moderate infection (100–399 eggs/gram) and heavy infection (≥ 400 eggs/gram) (**D**). The bar chart represents the percentage (%) prevalence with 95% confidence intervals (i.e., error bars). The dark (black) bars are the uninfected group and the light bars represent the infected group

*Schistosoma mansoni* infection prevalence was 25.2% (95% CI 20.5–30.2) with male participants having significantly high prevalence compared to their female counterparts (32.1% vs. 20.3%; *p* = 0.016) (Fig. 4A). Although the prevalence of 20.0%, 25.0% and 28.7% were recorded for age categories of 5–8 years, 9–12 years, and 13–17 years respectively, the increase was not statistically significant (Fig. 4B). The variation in the prevalence of *S. mansoni* infection across the school locations was significant (*p* = 0.001) as shown by Fig. 4C. Among the infected children, parasite load was considered light in 73.2% (60/82), moderate in 19.5% (16/82) and heavy infection in 7.3% (6/82) of the children (Fig. 4D). There was no significant difference in infection intensity by gender and age group (Table 3).

**Table 3:** *S. mansoni* egg load by gender and age group

	Light		Moderate		Heavy		p-value
	n	% (95%CI)	n	% (95%CI)	n	% (95%CI)	
Gender							
Male	33	76.7 (61.4–88.2)	7	16.3 (6.8–30.7)	3	7.0 (1.5–19.1)	0.72
Female	27	69.2 (52.4–83.0)	9	23.1 (11.1–39.3)	3	7.7 (1.6–20.9)	
Age group							
5–8 years	9	81.8 (48.2–97.7)	1	9.1 (0.2–41.3)	1	9.1 (0.2–41.3)	0.664
9–12 years	34	73.9 (58.9–85.7)	8	17.4 (7.8–31.4)	4	8.7 (0.2–20.8)	
13–17 years	17	68.0 (46.5–85.1)	7	28.0 (12.1–49.4)	1	4.0 (0.1–20.4)	

Chi-square test (χ^2^) was used to compare the proportions of categorical variables. n = number of participants, % = percentage, 95%CI = 95% confidence interval

### Activities in the stream are associated with *Schistosoma mansoni*

**Fig. 5:**
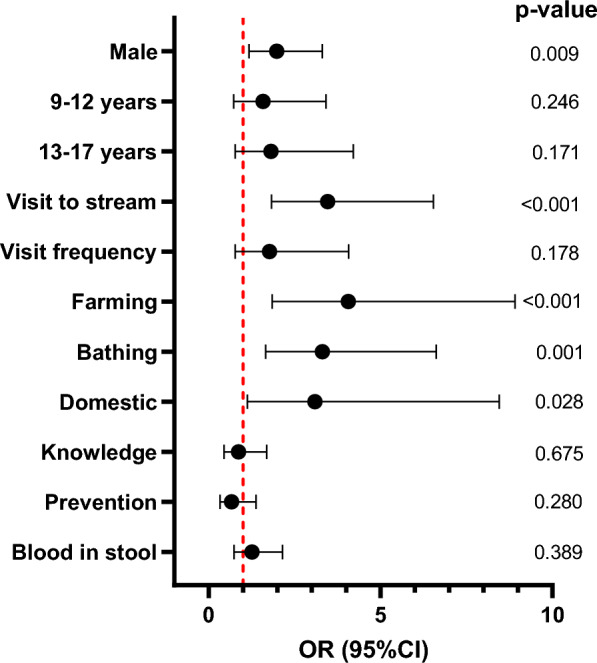
Predictors of *S. mansoni* infection. Association between *S. mansoni* infection and some demographic characteristics and childhood activities in streams. Odds ratio (OR), 95% confidence intervals (CI) and p values were calculated using separate multiple logistic regressing models adjusting for the different school’s children were sampled. The red vertical dotted line indicates no association between *S. mansoni* infection and the variables (OR = 1). Males and age groups were compared with females and children aged 5–8 years old respectively

Compared to girls, boys had 1.98 (95%: 1.18–3.31, p = 0.009) increased risk of being infected with *S. mansoni* (Fig. 5). The risk of *S. mansoni* infection increases among children aged 9–12 years (OR = 1.58 (95% CI 0.73–3.41, p = 0.246) and 13–17 years (odd ratio; OR = 1.81, 95% CI 0.77–4.21, p = 0.171) compared to those aged 5–8 years old. The differences were, however, not statistically significant. Children who visit the canal or dam were at 3.07 times (95% CI 1.59–5.91, p = 0.001) increased risk of being infected with *S. mansoni* compared to children who do not visit the stream. Childhood activities performed in the stream such as farming (OR = 3.88, 95% CI 1.75–8.57, p = 0.001), bathing or swimming in the stream (OR = 2.51, 95% CI 1.15–5.49, p = 0.021) and performing domestic chores (OR = 3.19, 95% CI 1.16–8.80, p = 0.025) all increased the risk of children getting infected with *S. mansoni*.

### Anaemia prevalence and severity

The average haemoglobin (Hb) concentration was 11.16 g/dl (SD: ± 1.39; range: 5.8–15.0). The proportion of anaemia cases among the children was 40.5% (95% CI 35.3–45.9); 40.2% were moderate and 0.3% were severe anaemia (Table 4). The prevalence of moderate anaemia was 43.3% among males and 38.0% among females (Table 4). The one child that had severe anaemia was a female. In general, the prevalence of anaemia was observed to decrease with increasing age groups from 54.5% among children aged between 5 to 8 years old to 38.0% among 9 to 12 years old and 35.6% among children aged between 13 to 17 years. However, there was no statistically significant difference between the age groups (*p* = 0.163). Moderate anaemia was detected across all the school communities except Nangalikinia where 1.4% of severe anaemia was detected. The prevalence of anaemia across the schools was not significantly different (*p* = 0.69).

**Table 4:** Top 10 citation count and degree of institution

	Normal		Moderate		Severe		p-value
	n	% (95%CI)	n	% (95%CI)	n	% (95%CI)	
Gender							
Male	76	56.7 (48.1–64.9)	58	43.3 (35.1–51.9)	0		0.46
Female	118	61.5 (54.3–68.1)	73	38.0 (31.4–45.1)	1	0.5 (0.1–3.6)	
Total	194	59.5 (54.1–64.7)	131	40.2 (35.0–45.6)	1	0.3 (0.0–2.2)	
Age group							
5–8 years	25	45.5 (32.6–58.9)	30	54.5 (41.1–67.4)	0		0.163
9–12 years	113	61.4 (54.1–68.2)	70	38.0 (31.3–45.3)	1	0.5 (0.1–3.8)	
13–17 years	56	64.4 (53.7–73.8)	31	35.6 (26.2–46.3)	0		
School/location							
Bonia	34	54.8 (42.2–66.9)	28	45.2 (33.1–57.8)	0		0.69
Gaani	40	58.0 (45.9–69.2)	29	42.0 (30.8–54.1)	0		
Korania	35	57.4 (44.5–69.3)	26	42.6 (30.7–55.5)	0		
Nangalikinia	44	63.8 (51.6–74.4)	24	34.8 (24.4–46.9)	1	1.4 (0.2–9.9)	
Tono	41	63.1 (50.6–74.1)	24	36.9 (5.9–49.4)	0		

Chi-square test (χ^2^) was used to compare the proportions of categorical variables

n, number of participants; %, percentage; 95%CI, 95% confidence interval

Anaemia was classified as normal (haemoglobin, Hb concentration ≥ 11.0 g/dl, moderate anaemia (Hb >7g/dl and <11.0 g/dl) and severe anaemia (Hb < 7 g/dl).

### Anaemia prevalence by infection status

**Fig. 6:**
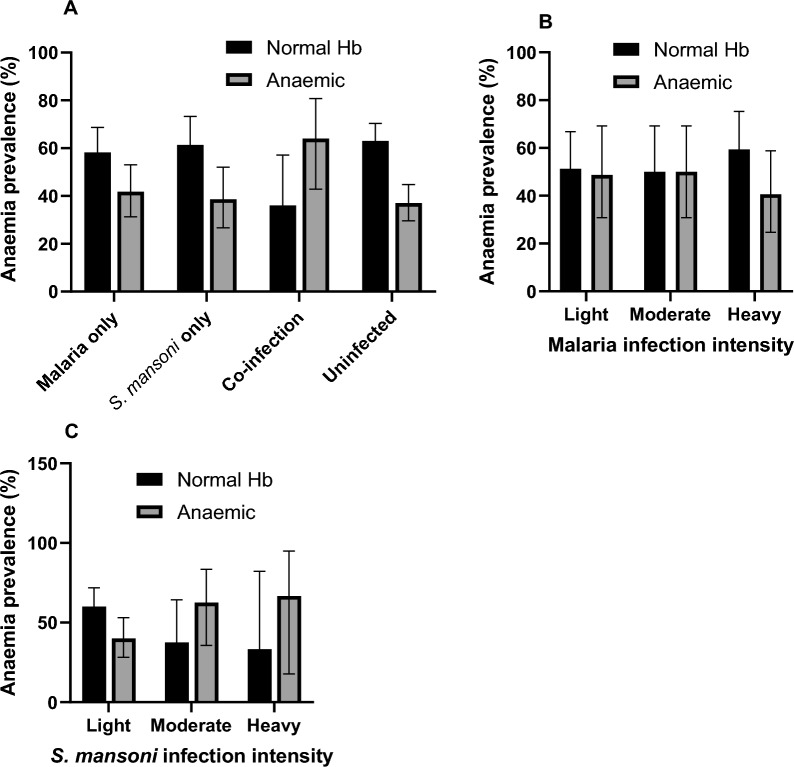
Distribution of anaemia among different infection groups and intensity. The prevalence of anaemia was stratified by infection status (**A**) and by the infection intensity of malaria (**B**) and *S. mansoni* (**C**). The infection intensity of malaria parasite load was classified as light infection (1–499 parasites/μl of blood), moderate infection (500–1999 parasites/μl of blood) and heavy infection (≥ 2000 parasites/μl of blood). The infection intensity of *S. mansoni* was classified as light infection (1–99 eggs/gram), moderate infection (100–399 eggs/gram) and heavy infection (≥ 400 eggs/gram). The bar chart represents the percentage (%) prevalence with 95% confidence intervals (i.e., error bars). The dark (black) bars normal haemoglobin (Hb) levels (Hb ≥ 11.0 g/dl) and the light bars represent the anaemic group (Hb > 7.0 and < 11 g/dl)

The overall prevalence of infection with any of the parasites (single or coinfection) was 49.4% (n = 161, 95% CI 44.0–54.8) with 7.7% (n = 25) coinfection. The prevalence of anaemia among children with no infection was 36.7% (95%CI 29.4–44.6) and 44.4% (95%: 36.5–52.4) among children with infection (malaria and/or schistosomiasis) (Fig. 6A). Anaemia among children with malaria infection only was 41.8% and 38.6% among children with *S. mansoni* infection only (Fig. 6A). Anaemia prevalence among children with co-infection of malaria parasite species and *S. mansoni* was 64.0%*.* There was no clear pattern observed with the prevalence of anaemia and malaria parasite density (Fig. 6B). The prevalence of anaemia increased with increasing *S. mansoni* egg load (Fig. 6C).

### Factors that affect haemoglobin levels

**Fig. 7:**
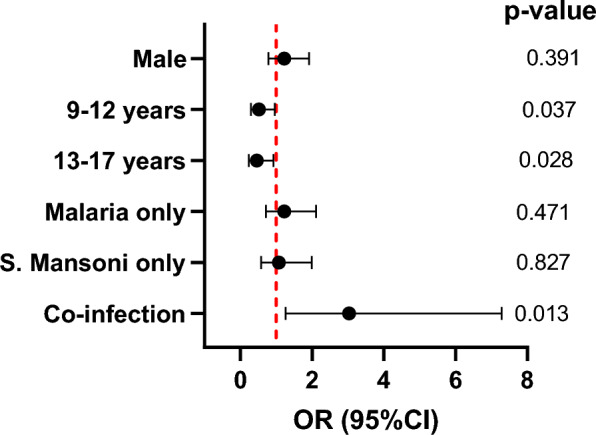
Predictors of anaemia among study participants. Association between anaemia status and sex, age group and infection status. Odds ratio (OR), 95% confidence intervals (CI) and p values were calculated using separate multiple logistic regressing models adjusting for the different school’s children were sampled. The red vertical dotted line indicates no association between anaemia and the variables (OR = 1). Males and age groups were compared with females and children aged 5–8 years old respectively

The risk of anaemia was 22% higher (OR = 1.22; 95% CI 0.78–1.91, p = 0.39) in males compared to females although it was not statistically significant (Fig. 7). There was a significantly reduced risk of anaemia among children aged 9–12 years (OR = 0.52, 95% CI 0.29–0.96, p = 0.037) and 13–17 years (OR = 0.46, 95% CI 0.23–0.92, p = 0.028) compared to those aged 5–8 years old. There was an increased risk of anaemia among children that were mono-infected with malaria parasite species (OR = 1.22, 95% CI 0.71–2.11, p = 0.47) or *S. mansoni* (OR = 1.07, 95% CI 0.58–1.99, p = 0.83) compared to those with no infection but this did not reach statistical significance. However, the risk of anaemia among children that were co-infected with the malaria parasite(s) and *S. mansoni* was 3.03 (95% CI 1.26–7.28, p = 0.013) compared to those with no infection.

## Discussion

This study presents findings of a cross-sectional survey of school children in five communities along the Tono irrigation area in the Kassena Nankana East Municipality of the Upper East Region, Northern Ghana. The findings indicate that (i) nearly half (49.4%) of the school children screened asymptomatically harboured at least one of the parasites or both (ii) malaria, intestinal schistosomiasis and anaemia were highly prevalent in the area (iii) comorbidity of malaria and intestinal schistosomiasis was common and (iv) comorbidity was associated with increased risk of anaemia among school children in the communities surveyed.

The area is hyper-endemic for malaria and one out of 3 (32.0%) children harboured the malaria parasites but were largely asymptomatic. This is similar to what was reported in the Kassena Nankana West District, an adjourning district in 2022 but differs from elsewhere in the country [9, 11, 42]. This is consistent with the variation in the malaria transmission intensity at the different ecological zones across Ghana. The differences in prevalence observed across the villages show the influence of micro-ecological determinants on malaria transmission in the area. It was common to find malaria parasite(s) in males compared to females. This could be due to the cultural perception of gender-specific roles that allow males to stay outdoor more often than females coupled with genetic, hormonal and immunological differences [43–45]. Characteristic of malaria, an increased rate of carrier status was observed with age group corroborating findings of previous studies in Ghana [11, 46–48]. Malaria immunity has been described as being age-dependent with older children and young adults more likely to harbour parasites without clinical symptoms. They, however, form a reservoir for continuous transmission and therefore, pose a threat to malaria control.

In addition to the asymptomatic carriage of malaria infections among the school children, over a quarter (25.2%) of the children were infected with *S. mansoni*. This is not surprising because open defecation was widely practiced and over 65% of them had access to stagnant water in the irrigation canals and reservoirs. This is coupled with a poor knowledge level of the disease. The area is endemic to *S. mansoni*. According to the World Health Organization, areas with *Schistosoma* infection ≥ 10% and < 50% are considered to be moderately endemic and require preventive control measures such as mass drug administration (MDA), water, sanitation and hygiene (WASH) and vector control [49]. In Ghana, MDA with praziquantel (PZQ) has been implemented since 2008 mainly among school-age children (SAC) for the control of the infection together with improved WASH practices [36]. MDA with PZQ was, however, interrupted during the 2020/2021 COVID-19 pandemic. At the time of this survey in September and October 2022, the children had not received their MDA with PZQ treatment. The prevalence of *S. mansoni* in this irrigated area was previously reported to be 54.2% in 2014 [16] suggesting nearly a 50% reduction compared to the current estimates. Males were at high risk of the infection consistent with previous studies in the area [9, 16]. Due to cultural and gender roles, more males visit the canals for farming and recreation purposes than females, activities which were both independently associated with increased risk of infection in the study. In the current study, 91.4% of the children reported practicing open defecation. These findings are corroborated by a WHO/UNICEF report on Ghana on WASH and water supply, respectively [50].

The prevalence of anaemia among the children was high with a number of them moderately anaemic. This is similar to other studies of similar age groups elsewhere [19, 20]. This could be due to the low parasite intensity observed in the study (73.2% were light infections), nutritional status as well as whether the infection was at the acute or chronic phase [20]. The study was conducted at a time of harvest when food was abundant in the area. The study observed that a single infection with malaria parasite(s) or *S. mansoni* infections did not independently increase the risk of anaemia significantly in the study populations similar to previous observation with malaria and *S. haematobium* in an adjourning district [9]. This finding is in contrast with other studies that have reported an increased risk of anaemia in children with only malaria and only *S. mansoni* infections [6, 21, 29, 51]. The contrasting results could be due to sample size since there was a trend towards an increased risk of anaemia, that did not reach statistical significance. Also, the differences between the current and previous studies could be due to geographic differences and changing patterns of both infections and anaemia. Another possible factor could be differences in the age profile of the participants of these studies. It was observed in the current study that older children were less likely to be anaemic compared to their younger counterparts. In addition, the aetiology of anaemia is multifactorial including age, genetics, infectious and non-infectious causes [21, 23, 46, 52–54]. Consistent with this assertion, in the current study, the only participant that had severe anaemia was neither infected with the malaria parasite nor *S. mansoni*. Notwithstanding the lack of association between mono-infections with malaria parasite or *S. mansoni*, children with concurrent infection of the asymptomatic malaria parasite(s) and *S. mansoni* were at three-time high risk of anaemia. This finding was corroborated by other studies that indicate that the interaction between multiple parasitic infections can increase the risk of anaemia [6, 17, 19–22]. The possible synergy between these two infections and anaemia is not elucidated. In animal models, pro-inflammatory cytokines such as tumour necrosis factor (TNF), interleukin-12 (IL-12), macrophage migration inhibitory factor (MIF) have been shown to lead to dyserythropoiesis, which subsequently results in anaemia [55–59]. The possible mechanisms of schistosome-induced anaemia include extra-corporeal loss resulting in iron deficiency, sequestration in the spleen, autoimmune haemolysis and inflammatory mechanisms [33]. Quite recently, an elevation of TNF was reported in individuals with *S. mansoni* infections [60].

Anaemia has effects on growth and development and cognitive abilities, which requires urgent attention [21, 27, 61, 62]. About half of the children were in the pre-pubertal age and therefore girls especially would soon start menses and if prompt control measures are not instituted, their anaemic situation would be aggravated.

Overall, the findings of this study reveal that asymptomatic carriage of malaria parasites and, *S. mansoni* infections were common among school children attending school in communities located along the Tono irrigation area. The study further revealed that anaemia was prevalent among school children mediated by concurrent infection of malaria parasites and *S. mansoni*. This suggests that children presenting with anaemia in this area may require more than just a malaria diagnosis to ensure children are free from the debilitating consequence of anaemia. The high level of anaemia among the children in this study is also suggestive of poor nutrition and poor health in the area. Therefore, in addition, MDA with PZQ campaigns should integrate malaria control and nutritional interventions to help reduce the current prevalence of asymptomatic malaria carriage and anaemia.

Key limitations of this study which investigated asymptomatic malaria parasite carriage, *S. mansoni* infection and anaemia include: (i) the effect of other parasitic infections such as soil-transmitted helminths (STH) that was not assessed; (ii) non-infectious aetiology of anaemia such as malnutrition was not evaluated; (iii) information on the cytokine profile of the infected participant was not assessed. Therefore, a firm conclusion could not be made on the correlation between concurrent malaria and *Schistosoma* infections and the risk of anaemia; (iv) the scope of the current study is limited to school children aged 5–17 years and may not be adequate for use to plan community interventions; (v) although malaria microscopy and Kato Katz techniques are the gold standards for malaria and *S. mansoni* diagnosis respectively, PCR technology could increase the sensitivity of detection since all the participants were asymptomatic and parasite load could be below the threshold for microscopy but PCR was not used due to resource constraints.

## Data Availability

The dataset used and/or analysed during the current study is available from the corresponding author on reasonable request.

## Abbreviations

Hb: Haemoglobin
KNEM: Kassena Nankana East Municipal
MDA: Mass drug administration
OR: Odd ratio
PZQ: Praziquantel
SD: Standard deviation
SAC: School-age-children
SSA: Sub–Saharan Africa
WASH: Water, sanitation and hygiene
